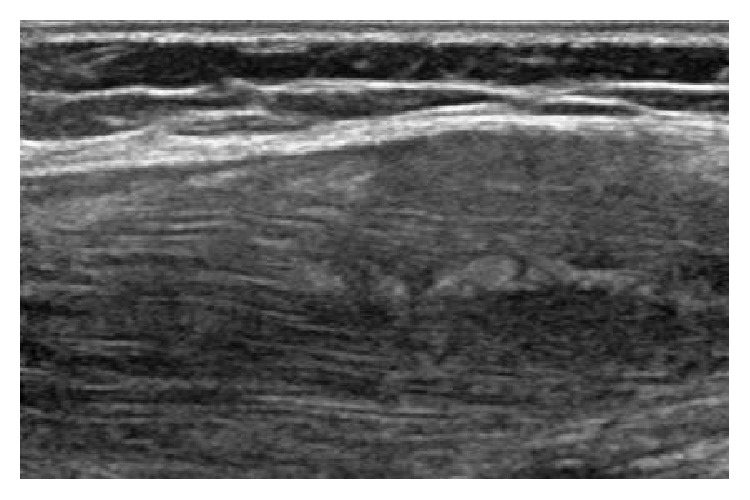# Postinjection Muscle Fibrosis from Lupron

**DOI:** 10.1155/2015/938264

**Published:** 2015-05-25

**Authors:** Erica Everest, Laurie A. Tsilianidis, Nouhad Raissouni, Tracy Ballock, Terra Blatnik, Anzar Haider, Douglas G. Rogers, B. Michelle Schweiger

**Affiliations:** ^1^Cleveland Clinic, 9500 Euclid Avenue, Cleveland, OH 44195, USA; ^2^Case Western Reserve University School of Medicine, 10900 Euclid Avenue, Cleveland, OH 44106, USA

## Abstract

We describe the case of a 6.5-year-old girl with central precocious puberty (CPP), which signifies the onset of secondary sexual characteristics before the age of eight in females and the age of nine in males as a result of stimulation of the hypothalamic-pituitary-gonadal axis. Her case is likely related to her adoption, as children who are adopted internationally have much higher rates of CPP. She had left breast development at Tanner Stage 2, adult body odor, and mildly advanced bone age. In order to halt puberty and maximize adult height, she was prescribed a gonadotropin releasing hormone analog, the first line treatment for CPP. She was administered Lupron (leuprolide acetate) Depot-Ped (3 months) intramuscularly. After her second injection, she developed swelling and muscle pain at the injection site on her right thigh. She also reported an impaired ability to walk. She was diagnosed with muscle fibrosis. This is the first reported case of muscle fibrosis resulting from Lupron injection.

## 1. Background

Lupron (leuprorelin acetate) is a gonadotropin releasing hormone analog (GnRHa). GnRH is normally released in a pulsatile manner, which can be interrupted by a constant serum level of an agonist. Resultant downregulation of the GnRH receptors reduces the amounts of estradiol and testosterone produced [1]. This medication is indicated for prostate cancer, endometriosis, uterine fibroids, and central precocious puberty, the condition of our patient [2]. GnRHas are the first-line treatment for central precocious puberty (CPP) [3].

Precocious puberty is early sexual maturation, before the age of eight in girls and the age of nine in boys [4]. There are two types: central (gonadotropin-dependent) and peripheral (gonadotropin-independent) types. CPP results from premature activation of the hypothalamic-pituitary-gonadal axis. CPP is concerning, because premature fusion of the epiphyseal growth plates decreases adult height and because early development may cause psychosocial issues [5, 6]. Interestingly, CPP is more prevalent in children who were adopted internationally, which is the case for our patient. These children are ten to twenty times more likely to develop precocious puberty. It is hypothesized that early nutritional deficits followed by rapid weight gain after adoption trigger the endocrine changes and physical growth of puberty prematurely [7, 8] However, the condition may still be idiopathic in nature, because idiopathic central precocious puberty makes up 90% of cases in females. Males are more likely to have pathological causes for central precocious puberty [1].

*Established Facts*

- Gonadotropin releasing hormone analogs are the standard of care for central precocious puberty [3].
- Injection site reactions are common from Lupron administration [9].

*Novel Insights*

- This is the first reported case of muscle fibrosis from a Lupron injection.

We report a case of a seven-year-old girl with muscle fibrosis of the right thigh following an injection of Lupron to treat her precocious puberty. A review of the literature reveals no previous reports of muscle fibrosis resulting from an intramuscular injection of Lupron.

## 2. Case Presentation

A six-year-eleven-month-old female presented to the Pediatric Endocrinology Clinic with signs of early puberty. Her mother noticed her daughter having adult body odor two months prior and development of her left breast (Tanner Stage 2) one month prior to the appointment. The patient denied any vaginal bleeding or discharge. She also denied pubic and axillary hair development, as well as any acne. Review of systems revealed increased thirst, while her physical exam was unremarkable. Her height and weight were at the 64th percentile for her age. This is an increase from the 14th percentile at the age of four, at which time she was adopted internationally. An X-ray to determine bone age was performed and showed bones between seven years ten months and eight years ten months of age by standards of Greulich and Pyle [10].

Laboratory data showed that the testosterone level was elevated at 12 ng/dL (0–9 ng/dL for Tanner Stage 1 females), and the hydroxyprogesterone level was elevated at 1.4 ng/mL (0.0–0.3 ng/mL). (A hydroxyprogesterone under 2 ng/mL and a testosterone that is not substantially higher than normal make congenital adrenal hyperplasia unlikely. However, the body odor suggested testing). The luteinizing hormone level of 2.0 mU/mL was consistent with CPP (diagnostic criteria is LH > 0.3 mU/mL). Follicle stimulating hormone was elevated at 4.5 mU/mL (0.0–4.0 mU/mL in prepubescent females) and estradiol 17B was also in the pubertal range at 39 pg/mL (<30 pg/mL). DHEA-S was normal at 28.6 ug/dL (0.0–37.0 ug/dL).

**Figure 1:**
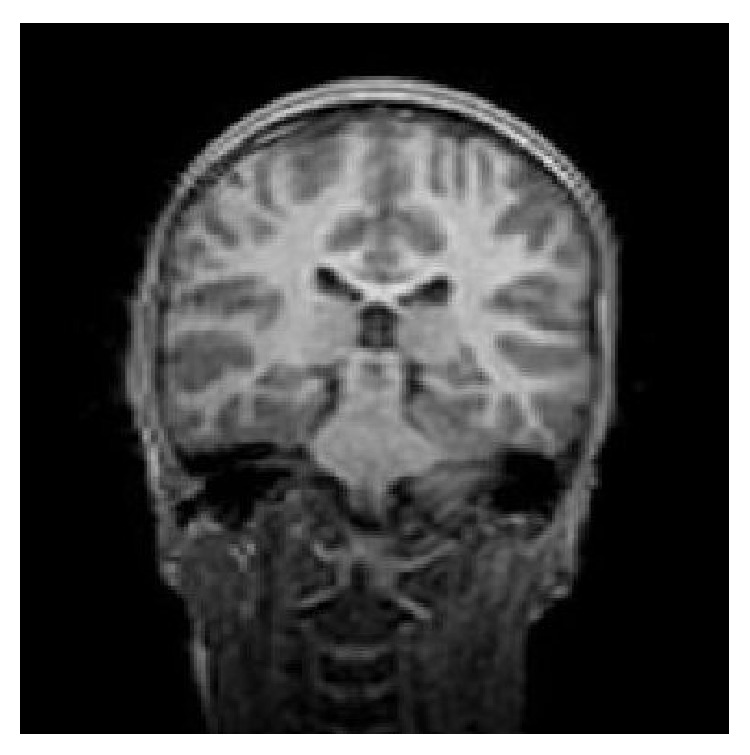


Given her increased growth velocity, thelarche, body odor, mildly advanced bone age, and gonadotropins and estradiol in pubertal ranges, she was diagnosed with CPP. She was given an MRI of her pituitary gland to rule out intracranial pathology as the cause of her precocious puberty (see Figure 1). The posterior T1 bright spot was present. The pituitary was homogenous in signal but was enlarged at 8 mm in the craniocaudal dimension. It had a convex superior margin, and no mass effect was noted. Overall, the pituitary gland was enlarged, but with no lesions to suggest an adenoma. We recommended injections of Lupron to halt puberty and prescribed Lupron Depot-Ped, 3 months 11.25 mg. We planned for her to return in five months, at which point she would have received two Lupron injections.

She received her first Lupron injection two weeks after the appointment. It was administered IM in the right vastus lateralis muscle. The patient tolerated the injection well, and she returned for a second injection in three months in the same location in her right thigh. At the time, the patient appeared to tolerate this injection well also. However, three weeks after the second injection, the patient had an appointment with a pediatric orthopedic surgeon. The reason for the visit was a lump on and swelling of her right thigh at the injection site. The lump measured 5 × 5 cm and was not tender to palpation. She reports pain in her right leg and trouble walking. She rated the pain as a 5 on a 0 to 10 scale with daily activities and with exercise. Her knee flexion was only fifteen degrees. Her right extremity showed no deformity. Ultrasound revealed no cellulitis or abnormal fluid collection at the injection site on her right thigh (see Figure 2). The lack of fluid indicated that this was not an abscess. She was diagnosed with muscle fibrosis based on the severe restriction of knee flexion due to lack of muscle excursion following the injection, as well as the presence of a mass at the injection site. She was instructed to have physical therapy.

## 3. Discussion

Muscle fibrosis and contracture following an intramuscular injection occurs most commonly in the anterior and lateral thigh [11]. Muscle fibrosis usually presents as atrophy, dimpling, reduced range of motion, and abnormal gait [12]. The major symptoms are recurrent dislocation of the patella and limitation of flexion of the knee. The contractures may form within weeks but also may take months to years after an injection [11]. These deformities may be coming later in our patient, since the diagnosis was merely weeks after the injection. For cases in which the diagnosis is made early or the fibrosis is mild, physical therapy and casting may be helpful [13]. However, these treatments are usually unhelpful in established cases. Surgery is often required and can create substantial improvement, especially when performed before there is permanent damage to the knee joint [14]. Since our patient was diagnosed quickly and began physical therapy, it is possible that this will prevent the muscle deformity from ever happening.

Thus far, there have been no reported cases of muscle fibrosis following an injection of Lupron. Treatment-related adverse effects occurred in 29% of patients dosed at 11.25 mg over three years in a long-term safety and efficacy study [9]. Common side effects at the injection site are pain and abscess, which occur in 5–15% of patients [5]. Also, there is the potential for acne, rash, blisters, facial swelling, weight gain, altered mood, headache, flushes and sweating, and vaginal symptoms, such as vaginitis, bleeding, and discharge [2].

The depot suspension of leuprolide acetate consists of microspheres of the drug within a biodegradable copolymer of lactic and glycolic acids [15]. With regard to the adverse effects of sterile abscess formation, it is believed that the cause is a reaction to the inert polymer rather than to the drug itself. The chance of having a reaction to these materials is estimated to be 3 to 13 per 100 children [16]. Given that patients have been successfully treated with the daily non-depot form of leuprolide acetate following an adverse reaction to the depot form, it seems as if the polymer was the component causing the problem [17]. However, there also have been cases reported of people with prostate cancer who have become resistant to GnRHa therapy following a sterile abscess formation [18, 19].

Although muscle fibrosis is a rare side effect of the Lupron depot injection, there are a substantial number of adverse effects reported at the injection site. Parents should be advised to monitor their child's injection site for abnormalities, even up to a few weeks or months afterward. Moreover, if they report any symptoms that resemble muscle fibrosis, they should be advised to seek medical care promptly.

## 4. Conclusion

This report describes the first case of muscle fibrosis of the thigh following an injection of Lupron Depot-Ped. The patient is a seven-year-old female being treated for central precocious puberty.

## Figures and Tables

**Figure 2:**